# Regional Ion Channel Gene Expression Heterogeneity and Ventricular Fibrillation Dynamics in Human Hearts

**DOI:** 10.1371/journal.pone.0082179

**Published:** 2014-01-10

**Authors:** Gopal Sivagangabalan, Hamed Nazzari, Olivier Bignolais, Ange Maguy, Patrice Naud, Talha Farid, Stéphane Massé, Nathalie Gaborit, Andras Varro, Krishnakumar Nair, Peter Backx, Edward Vigmond, Stanley Nattel, Sophie Demolombe, Kumaraswamy Nanthakumar

**Affiliations:** 1 Toronto General Hospital, Ontario, Canada; 2 INSERM, UMR915, l'institut du thorax, Nantes, France; 3 CNRS, ERL3147, Nantes, France; 4 Université de Nantes, Nantes, France; 5 University of Szeged and Division of Cardiovascular Pharmacology, Hungarian Academy of Sciences, Szeged, Hungary; 6 IHU LIRYC, Electrophysiology and Heart Modeling Institute, Pessac, France; 7 Lab IMB, University Bordeaux 1, Talence, France; 8 Montreal Heart Institute (MHI) and Université de Montréal Faculty of Medicine, Montreal, Canada; University of Minnesota, United States of America

## Abstract

**Rationale:**

Structural differences between ventricular regions may not be the sole determinant of local ventricular fibrillation (VF) dynamics and molecular remodeling may play a role.

**Objectives:**

To define regional ion channel expression in myopathic hearts compared to normal hearts, and correlate expression to regional VF dynamics.

**Methods and Results:**

High throughput real-time RT-PCR was used to quantify the expression patterns of 84 ion-channel, calcium cycling, connexin and related gene transcripts from sites in the LV, septum, and RV in 8 patients undergoing transplantation. An additional eight non-diseased donor human hearts served as controls. To relate local ion channel expression change to VF dynamics localized VF mapping was performed on the explanted myopathic hearts right adjacent to sampled regions. Compared to non-diseased ventricles, significant differences (p<0.05) were identified in the expression of 23 genes in the myopathic LV and 32 genes in the myopathic RV. Within the myopathic hearts significant regional (LV *vs* septum *vs* RV) expression differences were observed for 13 subunits: Nav1.1, Cx43, Ca3.1, Cavα2δ2, Cavβ2, HCN2, Na/K ATPase-1, CASQ1, CASQ2, RYR2, Kir2.3, Kir3.4, SUR2 (p<0.05). In a subset of genes we demonstrated differences in protein expression between control and myopathic hearts, which were concordant with the mRNA expression profiles for these genes. Variability in the expression of Cx43, hERG, Na^+^/K^+^ ATPase ß1 and Kir2.1 correlated to variability in local VF dynamics (p<0.001). To better understand the contribution of multiple ion channel changes on VF frequency, simulations of a human myocyte model were conducted. These simulations demonstrated the complex nature by which VF dynamics are regulated when multi-channel changes are occurring simultaneously, compared to known linear relationships.

**Conclusions:**

Ion channel expression profile in myopathic human hearts is significantly altered compared to normal hearts. Multi-channel ion changes influence VF dynamic in a complex manner not predicted by known single channel linear relationships.

## Introduction

The grave hemodynamic consequence of human ventricular fibrillation (VF) limits its study in vivo. Cell cultures [1] and animal models [2], [3], [4] have been utilized to test hypotheses regarding ionic currents and the spatiotemporal organization of VF. Although these models allow for elegant testing of the roles of single ion channels or currents [5], remodeling in myopathic human hearts involves multiple ion channels and requires a different investigative strategy. Regional differences in VF dynamics have mechanistic implications [2], [6]. In guinea pig hearts, a dominant high-frequency rotor in the left ventricle maintains VF, and appears to depend on inter-compartmental differences in I_K1_ [3]. Whether ion channel heterogeneity correlates with regional VF dynamics in human hearts has not been studied.

VF can be studied in explanted human hearts using a modified Langendorff perfusion system [7], [8]. This model provides the closest approximation to *in-vivo* VF in diseased hearts, allowing detailed electrophysiological mapping [9]. Our previous analyses in myopathic human hearts suggest that chamber-specific fibrillation dynamics are not entirely explained by regional structural differences [10]. An alternate hypothesis is that regional heterogeneity of ion channel expression may contribute to variations in VF dynamics. Regional transmural differences in the Ca^2+^ATPase [11] and Connexin [12] proteins as it pertains to conduction velocity and block with pacing in human hearts has been evaluated. These studies involved the testing of a hypothesis specific to a single factor in contribution to conduction in paced wedge sections, however regional multichannel correlation to VF in fibrillating whole human hearts has not been studied. We previously detailed the regional and tissue specific transcript signatures of ion channel genes in normal human hearts [13]. A comprehensive region-specific transcriptional expression profile of ion channels in myopathic human hearts and their potential relevance to spatial organization during VF may provide insight into potential therapeutic targets.

Therefore, we investigated comprehensive regional transcriptional differences in cardiac ion channel subunits between myopathic and non-diseased human hearts, and protein expression in a subset of important genes. We then tested the hypothesis that regional heterogeneity of ion channel transcripts will correlate with heterogeneity in local fibrillation dynamics.

## Materials and Methods

The experimental protocol was approved by the University Health Network ethics committee. Informed written consent was obtained from each patient and appropriate forms and documentation outlining the use of these myopathic human hearts and the purpose of this research study was provided to each patient. The University Health Network ethics committee approved the consent procedure.

The experimental protocol was approved by the Ethical Review Board of the Medical Center of the University of Szeged. Informed written consent was obtained for the use of these non-diseased human hearts in this research study.

All procedures conformed to the Helsinki Declaration of the World Medical Association.

### Myopathic Human Hearts

This experimental protocol was approved by the University Health Network ethics committee, and informed consent was obtained from each patient. Human cardiac tissue was dissected from eight cardiomyopathic patients (2 women, 6 men) who underwent cardiac transplantation. The mean age was 53±9 years, all patients had ejection fractions <20%. Immediately after explantation, hearts were immersed in cold Tyrode solution, and flushed thoroughly to remove blood particles. Left and right ventricular (LV and RV) and septal samples (1 mm thick) were dissected from the endocardial surfaces of the mid portion of the heart and snap-frozen with liquid nitrogen. As the study sought to determine the effects of myopathy induced ion channel remodeling on VF dynamics, as opposed to structural architecture effects, samples were taken from areas of healthy looking myocardium distant to areas of visual and palpable scar. We have previously demonstrated that ion channel activity is significantly different within hearts between areas of abnormal and relatively normal histology [14]. Summary of myopathic hearts used in the study are found in Table 1.

**Table 1:** Isolated Human Hearts Clinical Data.

#	Sex (M/F)	Age (Yrs)	Diagnosis	EF (%)	LV Dimensions Diastolic/Systolic (mm)	Medication prior to explant
1	F	48	ICM	<20	60/48	Digoxin, Spironolactone, Aspirin
2	M	60	ICM	<20	59/48	Furosemide, Metolazone, K-Dur, Ramipril
3	M	57	ICM	21	73/64	Carvedilol, Candesartan, Hydralazine
4	M	63	IDCM	<20	57/39	Metoprolol, Omeprazole, Sildenafil, Coumadin
5	F	47	IDCM	<20	78/64	Citalopram, Furosemide
6	M	61	ICM	13	78/73	Pravastatin, Furosemide, Glyburide, Aspirin
7	M	50	VCM	20	69/51	Digoxin, Aldactone, Ranitidine
8	M	36	ARVC	19	71/60	Sotalol, Amiodarone, Ramipril

IDCM - Idiopathic dilated Cardiomyopathy; ICM - Ischemic Cardiomyopathy; VCM – Valvular Cardiomyopathy; ARVC – Arrhtyhmogenic Right Ventricular Cardiomyopathy.

### Control Human Hearts

This experimental protocol was approved by the Ethical Review Board of the Medical Center of the University of Szeged. Eight (2 women, 6 men) non-diseased human hearts (age 45±8 years) were explanted from organ donors to collect pulmonary and aortic valves for transplant surgery. Tissue was collected and stored as described for the myopathic hearts.

### RNA preparation

Total RNA from each cardiac tissue was isolated and DNase-treated with the RNeasy Fibrous Tissue Mini Kit (Qiagen). The quality of total RNA was assessed by polyacrylamide-gel microelectrophoresis (Agilent 2100 Bioanalyser). Lack of genomic DNA contamination was verified by PCR.

### TaqMan real-time reverse transcriptase-polymerase chain reaction

The TaqMan Low-Density Array (TLDA, Applied Biosystems) technology was used in a two-step reverse-transcriptase-polymerase chain reaction process, as previously reported [13]. Briefly, first-strand cDNA was synthesized from 2 µg of total RNA using the High-Capacity cDNA Archive Kit (Applied Biosystems). PCR reactions were then performed on TLDA with the ABI Prism 7900HT Sequence Detection System (Applied Biosystems). The 384 wells of each card were preloaded with 96×4 predesigned fluorogenic TaqMan probes and primers (Table S1). Probes were labeled with the fluorescent reporter 6-carboxyfluorescein (FAM; Appler Corp.) at the 5′-end and with non-fluorescent quencher on the 3′-end. Data were collected with instrument spectral compensation with Applied Biosystems SDS 2.1 software and analyzed with the threshold cycle (C_t_) relative-quantification method. The genes selected for their cardiac expression encode 84 α- and β-ion channel subunits, Na^+^, K^+^-ATPase isoforms and proteins involved in calcium homeostasis and four reference genes for normalization.

For data normalization, we selected the Mean C_t_ [15] of the most expressed genes (Ct<30) and shifted those to the average one of HPRT. The mean C_t_ thus provides a reliable correction of the experimental variability increasing the biological variation accuracy [15]. The relative expression level of each gene is indicated as the 2^−ΔCt^. In order to determine regional specific heterogeneity in transcriptional expression in the myopathic human hearts, the relative expression levels of each gene and for each region were averaged and compared between LV, RV, and septum. Outliers were elicited and removed from the mean of each group after a Grubbs' test was applied.

### Protein extraction and Western-blot

Freshly-isolated left ventricular endocardial samples from normal and myopathic hearts were fast-frozen in liquid nitrogen, pulverized and homogenized in TNE-buffer containing: Tris 25-mmol/L, EDTA 5-mmol/L, EGTA 5-mmol/L, NaCl 150-mmol/L, NaF 20-mmol/L, Na_3_VO_4_ 0.2-mmol/L, ß-glycerophosphate 20-mmol/L, AEBSF 0.1-mmol/L, leupeptin 25-µg/mL, aprotinin 10-µg/mL, pepstatin 1-µg/mL, microcystin-LR 1-µmol/L; pH 7.34, HCl. Homogenized samples were centrifuged at 1,000 g for 10 minutes, supernatant collected and ultracentrifuged at 100,000 g for 1 hour. The supernatant was resuspended and incubated in TNE-buffer containing 1% Triton-X100. Protein concentration was determined by Bradford assay (Biorad). All steps were carried out on ice at 4–5°C. Protein samples (20 µg) were separated on 8% poly-acrylamide SDS-PAGE and transferred electrophoretically onto PVDF membranes. The PVDF membranes were blocked in a PBS-solution containing 0.05% (v/v) Tween-20 and 5% (w/v) nonfat dried milk (NDM) and incubated overnight at 4°C with primary antibodies diluted in PBS containing 0.05% Tween-20 and 1%-NDM. After washing with PBS-Tween solution/1%-NDM, membranes were hybridized with HRP-conjugated secondary antibody. Immunoreactive bands were detected by enhanced chemiluminescence using BioMax MS/MR films. Protein quantification was performed with the Quantity One® software (Biorad). All of the expression data are provided relative to GAPDH staining for the same samples on the same gels.

### Antibodies

Primary antibodies (1/2000) included monoclonal mouse anti-SERCA2 ATPase (2A7-A1; MA3-919), monoclonal mouse anti-Phospholamban (2D12; MA3-922), polyclonal rabbit anti-Calsequestrin (PA1-913) and monoclonal mouse anti-Na^+^,K^+^-ATPase alpha-3 (XVIF9-G10; MA3-915) from Thermo scientific, monoclonal mouse anti-Kir2.2 (S24-1; ab84821) from Abcam, monoclonal mouse anti-Kir2.3 (N25/35; 75-069) from Neuromab, and monoclonal anti-GAPDH (10R-G109a) from Fitzgerald. Peroxidase-conjugated AffiniPure goat anti-rabbit IgG (111-035-144) and Affinipure donkey anti-mouse IgG (715-035-151) from Jackson ImmunoResearch were used as secondary antibodies (1/10,000).

### Human Langendorff- Cardiac Electrophysiology

After the samples were removed from the myopathic hearts the coronary arteries were selectively cannulated and flushed thoroughly with Tyrode solution (composition: 118.1 mM NaCl, 4.7 mM KCl, 2.5 mM CaCl_2_, 1.2 mM MgSO_4_, 24.9 mM NaHCO_3_, 1.2 mM KH_2_PO_4_, 6.1 mM glucose). The Tyrode was oxygenated with a pediatric oxygenator connected to a carbogen (95% O_2_, 5% Co_2_) cylinder. The flow was subsequently maintained at 0.9 to 1.1 ml/g/min at 38°C, and the hearts were placed in a heat-jacketed reservoir at 38°C. Electrical mapping was performed on the epicardium using a custom sock array and on the LV endocardium using a second balloon array as described previously [16]. Due to the marked heterogeneity of VF over short intra-ventricular distances, recordings were made from three electrodes adjacent to tissue sampling locations, allowing local electrophysiological correlation with local gene expression. VF was induced within 20 minutes of cannulation by placing a 9-volt battery on the epicardium. Bipolar electrograms were evaluated to ensure appropriate contact. After data acquisition, all electrograms were low-pass filtered at 60 Hz using a 112^th^ order FIR equiripple filter, and down-sampled to 125 Hz. For analysis, 20-second segments of VF were included, from the septum, RV and LV free walls.

### Fibrillation Dynamics

To determine fibrillation dynamics right adjacent to the region sampled we used the most robust of the near field VF dynamic. Two local parameters of local fibrillation dynamics were assessed; cycle length (activation rate) and conduction block. Local activation rates were calculated using both unipolar and bipolar electrograms at the local recording sites. Peak dV/dt in the unipolar signal was used as a marker of local activation, which was confirmed to coincide with the activation in the bipolar signal. Local cycle length (interval between activations) was measured over 5 seconds and the values averaged to estimate the cycle length for that region. To estimate spatial organization we used double peak incidence (DPI) as described by Evans *et al.* [17] Double spectral peaks have been shown to be an indicator of conduction block. We computed the fraction of total time the DPI were present in an electrogram and averaged it over 3 electrodes in a region to get the mean fraction of time the DPI was present per VF episode.

### Regional comparison of transcripts

Analysis of variance was used to compare mean values between myopathic and normal heart ventricular transcript expression, both LV and RV comparisons. Comparisons of gene expression between ventricular regions were conducted within sets of samples from individual patients, thus controlling for inter-individual variability. Comparisons of gene expression within myopathic heart chambers (LV vs RV vs Septum) was performed with a general linear model, each gene entered as a dependent variable, region entered as the fixed factor, and individual heart identification included in the model. Corrections using LSD were used for paired comparisons. Significance was evaluated for P<0.05.

### Correlating ion-channel transcriptional profiling to electrophysiological measures

Local transcriptomal profiling was correlated to VF parameters by regression analysis. To limit the number of variables entered in regression analysis, we entered only genes that are functionally important in the heart (34/84). The independent predictors of VF cycle length and conduction block (DPI) were determined by multiple regression including stepwise selection. The independent variables were entered one at a time in the order in which they most improved the model R^2^. The alpha level for retention of a variable in the model was 0.15.

### Computer Simulations

Computer simulations were performed using the ten Tusscher human epicardial ventricular ionic model [PMID:16565318] as a basis. Myopathic versions for the left and right ventricles were created by modifying the simulation parameters of the model according to protein expressions levels (Table 2). Monodomain simulations on a 12×5 cm sheet with sealed edges were carried out using the CARP simulator [PMID:14716595]. The temporal discretization used was 25 microseconds while the spatial discretization was set at 150 micrometers. A stable rotor was created, using the unmodified ionic model, by applying an S1 along the left edge of the tissue and applying an S2, which, covered the lower left quarter of the sheet. The rotor was allowed to stabilize and the state saved after 1.5 seconds, which became the initial conditions for all subsequent simulations. Modifications were made to the ionic model and then the activity was continued for another four seconds. All rotors persisted for this length of time. The average dominant frequency of these four seconds of activity was determined by taking discrete Fourier transforms of each node in the model, selecting the frequency associated with the highest energy component, and then averaging these frequencies over the whole sheet.

**Table 2:** Relative changes in ionic model parameters compared to published normal values.

Parameter	LV myopathic	RV myopathic
Cx43	0.8	1.15
IKr	0.55	0.6
NaK	0.66	0.8
IK1	1.64	1.6
NCX	1	1.2
SERCA	0.5	0.5
CASQ	1.25	1.4

## Results

### Clinical characteristics

Table 1 summarizes clinical characteristics of myopathic heart patients.

### Ion channel transcript signatures in myopathic human hearts compared to normal human hearts

Expression levels for the myopathic hearts and normal hearts were compared by ventricular chamber. The genes showing significant differences (p<0.05) in expression are presented for the LV (Tables 3 and 4) and RV (Tables 5 and 6). The expression profile of all genes analyzed in this study can be found in Table S2.

**Table 3:** Comparison of Na^+^, Cl^−^ and Ca^2+^ channel subunits, Ca^2+^ handling proteins and exchanger expression levels between myopathic and normal LV's.

Subunit	Normal	Myopathic	P-value
Nav1.3	3.51±0.78	6.80±1.02	0.022
Nav2	220.88±22.41	380.50±53.26	0.015
Navβ2	33.40±8.42	74.81±3.48	<0.001
Cav3.2	3.49±0.26	8.08±1.51	0.010
Cavα2δ1	159.86±15.38	236.21±15.84	0.004
CFTR	2.27±0.64	0.15±0.04	0.015
Na/K ATPase α3	1599.52±146.13	1128.90±70.86	0.012
Na/K ATPase β1	1119.32±74.20	836.58±30.03	0.003
SERCA2	4635.35±333.73	2214.78±160.50	<0.001
PLN	16292.37±1380.77	8658.84±460.77	<0.001
CASQ2	2108.07±90.71	2671.54±121.65	0.002

Units for all values are 2^−ΔCt^ versus reference gene (×100), expressed as mean ± SEM. Only expression comparisons achieving p<0.05 are presented.

**Table 4:** Comparison of K^+^ channel subunit expression levels between myopathic and normal LV's.

Subunit	Normal	Myopathic	P-Value
Kv1.4	11.64±1.82	21.19±1.20	0.001
Kv1.5	12.55±1.18	17.99±1.63	0.017
Kv1.7	5.40±1.27	0.81±0.22	0.033
Kv3.4	10.66±1.19	17.23±1.36	0.003
Kv4.3	26.72±1.60	16.30±0.91	<0.001
Kv11.1/HERG	191.23±17.96	105.68±5.08	<0.001
Kir2.2	74.62±8.69	46.42±2.16	0.007
Kir2.3	80.89±10.26	132.44±12.81	0.007
Kir3.4	37.04±5.87	10.17±2.01	0.001
Kvβ1	9.53±1.53	19.19±1.87	0.001
KCHIP2	222.26±33.29	47.73±11.61	<0.001
SUR1	4.11±0.49	7.92±1.66	0.045

Units for all values are 2^−ΔCt^ versus reference gene (×100), expressed as mean ± SEM. Only expression comparisons achieving p<0.05 are presented.

**Table 5:** Comparison of Na^+^, Cl^−^ and Ca^2+^ channel subunits, Ca^2+^ handling proteins and exchanger expression levels between myopathic and normal RV's.

Subunit	Normal	Myopathic	P-Value
Nav1.3	3.84±0.89	6.73±0.67	0.013
Nav1.7	6.56±0.67	4.04±0.42	0.007
Navβ2	27.00±6.45	61.77±4.40	0.001
Cavα2δ1	131.48±8.85	216.61±14.74	<0.001
CIC-7	93.32±13.89	53.16±3.85	0.015
CFTR	2.48±0.53	0.32±0.14	0.005
HCN3	4.43±0.67	2.75±0.21	0.031
HCN4	73.05±9.05	43.08±7.48	0.023
Na/K ATPase α3	1769.95±97.85	1277.98±91.14	0.002
NCX1	625.55±49.09	762.10±25.93	0.028
SERCA2	4827.01±306.21	2863.62±253.89	<0.001
SERCA3	15.92±1.48	11.32±1.42	0.042
PLN	14969.11±2155.61	9954.70±593.50	0.042
CASQ2	2032.56±84.64	2809.94±170.95	0.001
ITPR1	76.11±5.92	55.36±4.40	0.014

Units for all values are 2^−ΔCt^ versus reference gene (×100), expressed as mean ± SEM. Only expression comparisons achieving p<0.05 are presented.

**Table 6:** Comparison of K^+^ channel subunit expression levels between myopathic and normal RV's.

Subunit	Normal	Myopathic	P-Value
Kv1.4	8.43±0.87	20.35±2.32	<0.001
Kv1.7	5.77±1.25	0.69±0.33	0.002
Kv3.3	2.18±0.24	1.52±0.16	0.038
Kv4.3	26.07±1.73	18.17±1.62	0.005
Kv11.1/HERG	195.47±14.86	115.55±3.47	<0.001
KvLQT1	91.47±3.23	63.01±3.23	0.001
TWIK-1	80.93±8.82	52.64±7.18	0.026
Kir2.1	95.99±12.98	169.16±29.44	0.004
Kir2.2	90.23±9.83	53.12±2.47	0.039
Kir2.3	46.48±6.30	84.15±8.27	0.003
Kir3.4	20.90±13.92	12.32±2.22	0.036
Kir6.2	41.44±6.10	27.26±2.26	0.047
Kvβ1	10.66±1.04	16.91±1.50	0.004
KCHIP2	320.57±22.74	119.68±25.22	<0.001
TASK2	2.27±0.47	0.97±0.18	0.021
MinK	11.80±0.99	16.82±1.83	0.003
SUR1	3.84±0.84	8.26±1.57	0.026

Units for all values are 2^−ΔCt^ versus reference gene (×100), expressed as mean ± SEM. Only expression comparisons achieving p<0.05 are presented.

#### Calcium handling proteins

In the myopathic ventricles the expression of SERCA2 and its regulatory protein PLN were significantly reduced compared to the normal hearts. Expression of the sarcoplasmic reticulum (SR) Ca binding protein calsequestrin was significantly increased in both myopathic ventricles.

#### Calcium Channel subunits

In the myopathic LV, expression levels of the α1 subunit Cav3.2 and the α2δ subunit Cavα2δ1 were higher than control LVs. In the myopathic RVs expression of Cavα2δ1 was higher than controls.

#### Exchangers

In the myopathic LV and RV the expression of Na^+^/K^+^ ATPase α3 was significantly reduced compared to the normal cohort.

#### Sodium Channel subunits and Connexins

Expression for Nav1.3, Nav2, and Navβ2 was significantly higher in the LV of the myopathic hearts compared to normal hearts, while, in the myopathic RV, expression was higher for Nav1.3 and Navβ2, and lower for Nav1.7 than controls. No significant difference was observed for Cx40, Cx43, or Cx45 transcript expression for either ventricle between the myopathic and normal hearts.

#### Potassium channel subunits

Transcripts responsible for transient inward current I_to_ were altered in an inconsistent pattern in the myopathic hearts. In the LV, Kv1.7 and Kv4.3 expression was reduced, while Kv3.4 expression was increased. In the RV Kv1.4 expression was increased, while expression of Kv1.7 and Kv4.3 was decreased. Transcripts responsible for the inward rectifying current I_K1_, Kir2.1 and Kir2.2, showed differing expression profiles between LV and RV when compared to the normal ventricles. Kir2.2 was significantly decreased in both LV and RV, while Kir2.1was significantly upregulated in the RV. The expression of Kir3.4 transcripts, which contribute towards the production of the inward rectifying current I_kACh_, was significantly weaker in the myopathic ventricles than the normal ventricles. The α- (Kir6.1 and Kir6.2) and ß-subunits (SUR1 and SUR2) responsible for the production of I_kATP_ were also analyzed. Of these subunits SUR1 showed increased expression in both myopathic ventricles, while the Kir6.2 showed decreased expression in myopathic RV when compared to normal ventricles.

### Regional ion channel transcript profile in myopathic human hearts

**Figure 1:**
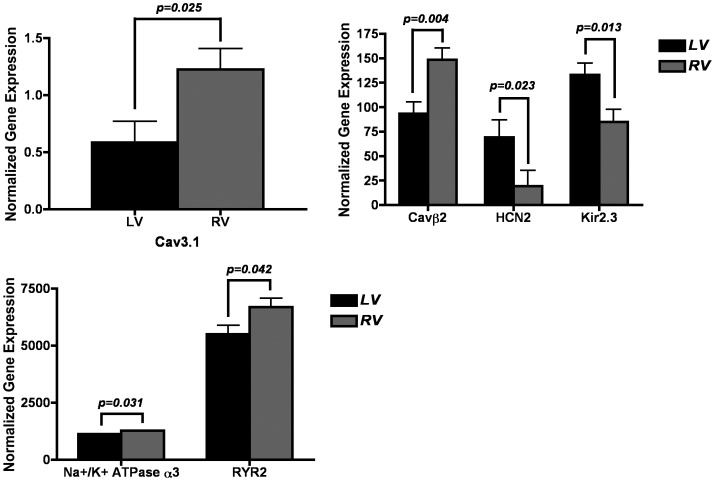
Significant (p<0.05) differences in gene expression levels between LV and RV samples within myopathic hearts. Differentially expressed genes in LV versus RV. Units for all expression values are 2^−ΔCt^ versus reference gene (×100). Only expressions comparisons achieving p<0.05 are depicted.

**Figure 2:**
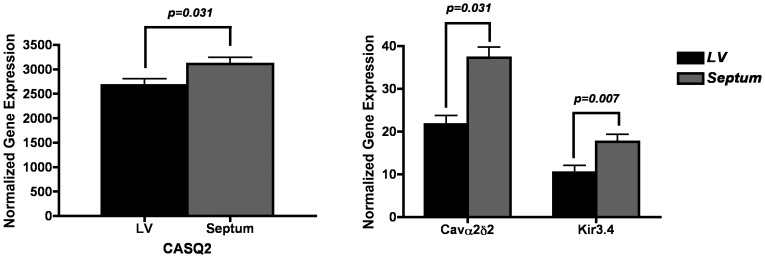
Significant (p<0.05) differences in gene expression levels between LV and septal samples within myopathic hearts. Differentially expressed genes in LV versus septum. Units for all expression values are 2^−ΔCt^ versus reference gene (×100). Only expressions comparisons achieving p<0.05 are depicted.

**Figure 3:**
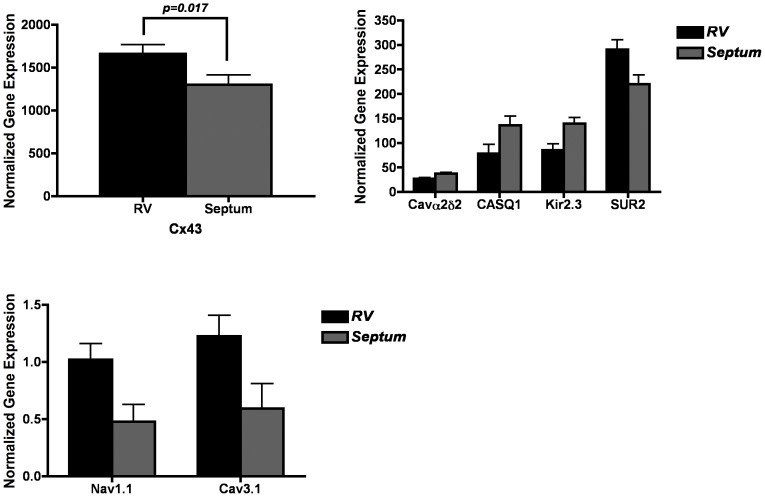
Significant (p<0.05) differences in gene expression levels between RV and septal samples within myopathic hearts. Differentially expressed genes in RV versus septum. Units for all expression values are 2^−ΔCt^ versus reference gene (×100). Only expressions comparisons achieving p<0.05 are depicted.

We compared expression between the LV, RV and septum within the 8 myopathic hearts. Figures 1–3 show paired comparisons between chambers for the transcripts that demonstrate significant difference, p<0.05 (Figure 1 LV *vs* RV; Figure 2 LV *vs* septum; Figure 3 RV *vs* septum).

#### Calcium channels and calcium handling proteins

Expression of Cav3.1 was significantly higher in the RV compared to the septum and LV. RyR expression was much higher in the RV than the LV. Calsequestrin expression was significantly higher in the septum. The expression of the Cavß2 showed higher levels of expression within the RV compared to the LV, while the Cavα2δ2 was higher in the septum compared to both the LV and RV.

#### Sodium, chloride, connexins and pumps

The expression of Cx43 was greater in the RV than the LV or the septum. Expression of Na^+^/K^+^ ATPase α3 was greater in the RV than the LV and septum.

#### Potassium channels

Kir2.3 (I_k1_) expression was significantly greater in the LV and septum compared to the RV. Kir3.4 (I_kACh_) expression was significantly stronger in the septum compared to the LV. Expression for the pacemaker channel HCN2 was much stronger in the LV than the RV. SUR2 expression was significantly higher in the RV compared to the septum.

### Transcriptomal Correlation to VF Heterogeneity

**Figure 4:**
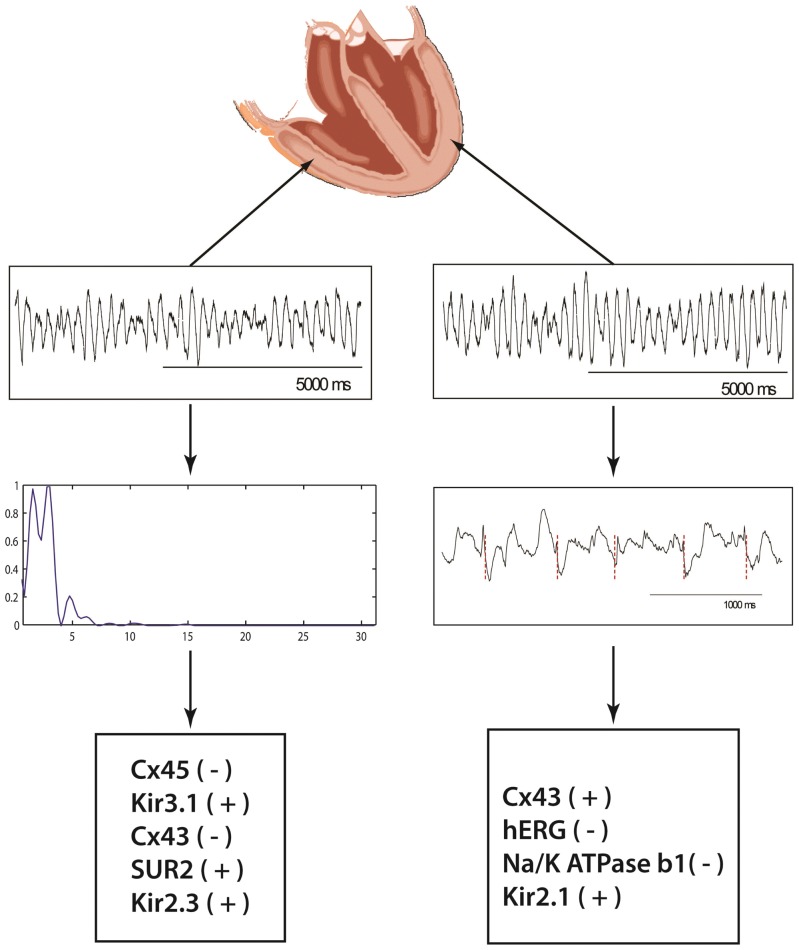
Regional VF Mapping and determination of local activation rate and conduction block. Local unipolar recordings of VF are displayed from the LV and RV. For the LV recording unipolar activation is marked (maximal negative dV/dt) demonstrating local activation rate and cycle length. The FFT of the RV recording shows 2 peaks indicating local conduction block. The bottom panels show the determinants of conduction block (left) and cycle length (right) in the order they entered the regression models.

Mean VF cycle length was 313 ms (LV = 239 ms; RV = 384 ms; Septum = 308 ms, p = NS). Fraction of time DPI was present was in average 25.7% (LV = 31.4%; RV = 20.2%; Septum = 25.4%. p = NS). Figure 4 demonstrates the local VF unipolar electrogram recordings and examples of local activation rate and spectral analysis. The results of the regression analysis are presented in Table 7. Amongst the 34 transcripts analyzed, variability in Cx43, hERG, Na^+^/K^+^ ATPase β1 and Kir2.1 expression correlated with variability in VF cycle length (p<0.001). Cx43, Kir2.1 had a positive slope, indicating increasing expression levels contributed to increasing cycle length, while hERG and Na^+^/K^+^ ATPase β1 had a negative slope, indicating increasing expression levels correlated with decreasing cycle length. Of the 34 transcripts in the model, variability in Cx45, Kir3.1, Cx43, SUR2, and Kir2.3 correlated with variability in conduction block (DPI) incidence during VF. Kir3.1, Kir2.3 and SUR2 had a positive slope, indicating increasing expression levels correlate with increasing conduction block, while Cx45and Cx43 had a negative slope, indicating that increasing expression levels correlate with decreasing conduction block.

**Table 7:** Results of regression analysis for predictors of VF cycle length and conduction block (DPI) incidence.

Stepwise Multiple Regression (for predicting Cycle Length and incidence of Conduction Block)			
	Model R^2^	Regression Coefficient	p-Value
Predictor Variables* (For Cycle Length)			
Cx43	0.57	0.415	<0.001
Na^+^/K^+^ ATPase β1	0.79	−0.428	0.090
Kir2.1	0.86	8.82	0.044
hERG	0.95	−7.95	0.004
Predictor Variables** (For Conduction Block)			
Cx45	0.41	−0.59	0.001
Kir3.1	0.63	2.24	0.068
Cx43	0.72	−0.03	0.006
SUR2	0.79	0.0197	0.014
Kir2.3	0.88	0.19	0.049

Stepwise multiple regression analysis was performed to evaluate the contribution of ion channels to Cycle length or incidence of conduction block. Expression level of each ion channel was added to the regression model and those with significant contribution to dependent variables were retained in the model. Data presented in the table demonstrates the most appropriate fit, capable of predicting the incidence of CL and conduction block.

Among all ion channels assessed, expression of Cx43, Na/K ATPase β1, Kir2.1 and hERG were the most significant predictors of Cycle length.

Among all ion channels assessed, expression of Cx45, Kir3.1, Cx43, SUR2 and Kir2.3 significantly correlated with incidence of conduction block.

### Protein analysis

**Figure 5:**
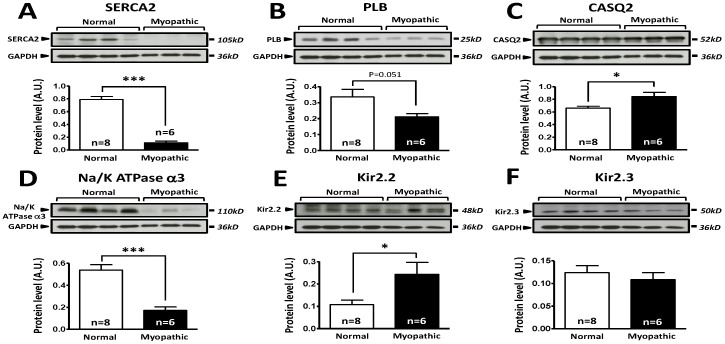
Comparison of protein expression in LV sample from control and cardiomyopathic patients. A: Top; representative SERCA2 and respective GAPDH stainings obtained in normal and cardiomyopathic LV samples. Bottom; Mean±SEM, *** P<0.001. B: Top; representative phospholamban (PLB) and respective GAPDH stainings obtained in normal and cardiomyopathic LV samples. Bottom; Mean±SEM, P = 0.051. C: Top; representative calsequestrin 2 (CSQ) and respective GAPDH stainings obtained in normal and cardiomyopathic LV samples. Bottom; Mean±SEM, * P<0.05. D: Top; representative Na/K-ATPase-α3 and respective GAPDH stainings obtained in normal and cardiomyopathic LV samples. Bottom; Mean±SEM, *** P<0.001. E: Top; representative Kir2.2 and respective GAPDH stainings obtained in normal and cardiomyopathic LV samples. Bottom; Mean±SEM, * P<0.05. F: Top; representative Kir2.3 and respective GAPDH stainings obtained in normal and cardiomyopathic LV samples. Bottom; Mean±SEM. n = 8 control LV samples, n = 6 cardiomyopathic LV samples.

We determined the protein expression levels for a subset of genes with Western blot analysis of freshly isolated LV endocardial tissue from control and myopathic hearts (Figure 5). Of the genes that were analyzed, SERCA2, PLB, CASQ2 and Na^+^/K^+^ATPase α3 (Figure 5A–D) demonstrated differences in protein expression between control and myopathic hearts, which were concordant with the mRNA expression profiles for these genes. For each of the genes, protein expression was significantly reduced in myopathic hearts when compared to controls, with the exception of PLB, which was not significantly different but did show a decreasing trend. In the case of Kir2.2, protein expression levels are elevated in myopathic hearts versus controls (Figure 5E), in contrast to the mRNA expression profile of this channel. In the case of Kir2.3 no significant difference was observed between myopathic and control LV endocardium (Figure 5F). The discordance between the protein and mRNA expression profiles for Kir2.2 and Kir2.3 may be attributed to translational and post-translational regulation specific processes.

### Computer simulations

**Figure 6:**
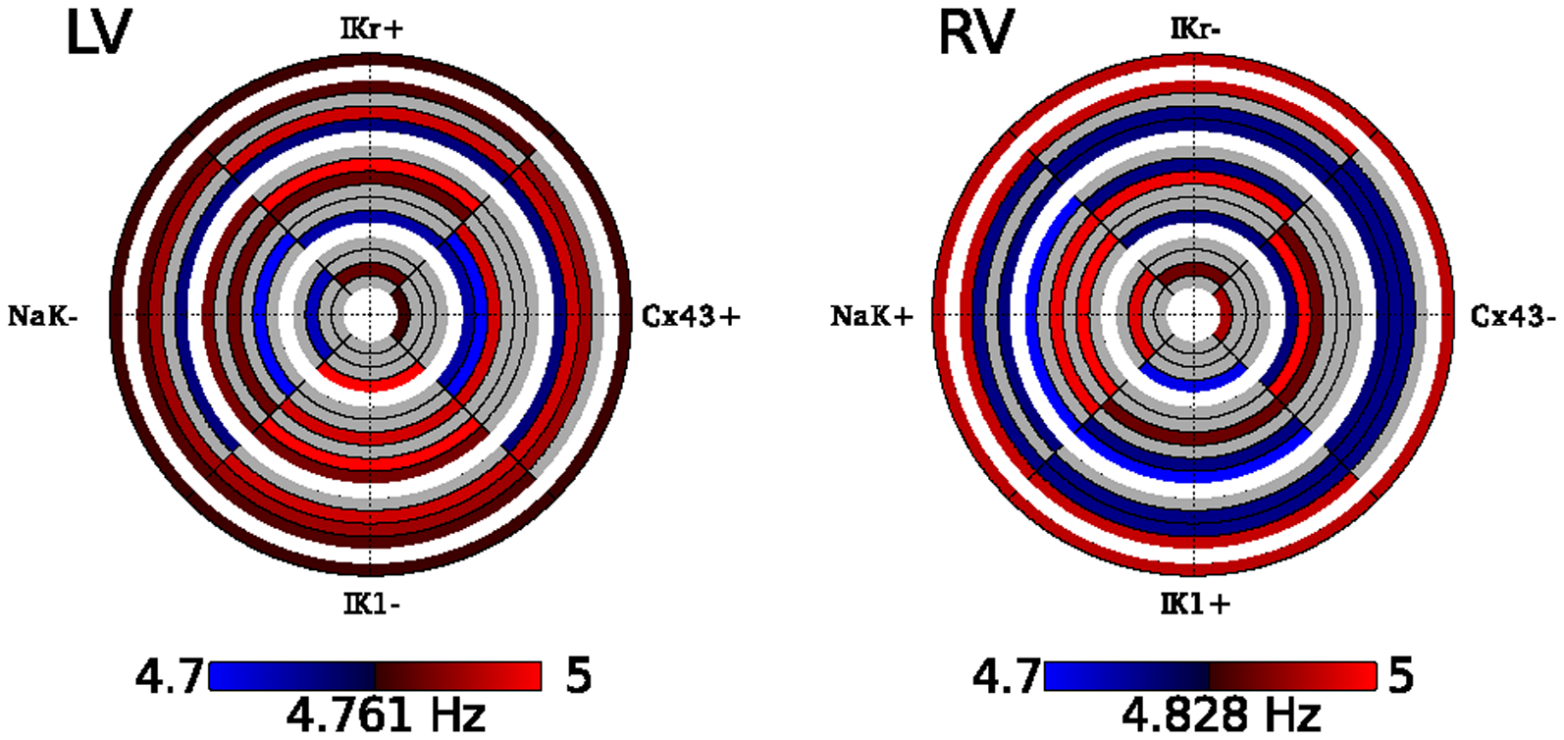
Computer simulations. Effect of protein level changes on average frequency. Starting with the left (A) or right (B) ventricular myopathic ionic model, parameters were changed to match expression levels in the other ventricle as specified in Table 2. Increases in frequency are red while decreases are blue, with brighter colors indicating more change from control. Each ring represents a simulation with each quarter circle representing a parameter change. If the parameter was altered for the simulation, it is colored coded according to the resultant frequency change, otherwise it is left as grey. Frequencies range from 4.71–4.99 Hz with a baseline frequency of 4.83 Hz for the LV and 4.76 Hz for the RV.

Using the ventricular myocyte model described in the methods section, we manipulated only the parameters of the model which were associated with frequency changes to see their effect in isolation and in combination with each other, using protein expression levels derived from our study (Table 2). Thus, NCX, CASQ, and SERCA were set differently for LV and RV myopathic tissue but were not varied to see their effect on frequency. The modeling experiments yielded a stable rotor and subsequently parameters were manipulated to determine the effects on VF frequency. Starting with the protein levels for one ventricle, either myopathic LV or RV, the four parameters were systematically changed to those of the other ventricle. The results of these simulations are summarized as circular data plots (Figure 6).

The circular plots represent the changes in rotor frequency after manipulation of four different molecular components: Cx43, hERG, Na^+^/K^+^ ATPase ß1 and Kir2.1. Within the data plots, red indicates an increase and blue a decrease in cycle frequency, with color intensity corresponding to the degree of change. Examining the plots radially from the inner to outer circles illustrates the contribution of each molecular component to cycle frequency as the number of components changed was increased. The only component, which showed a consistent response across all changes, was IK1, which was associated with an increase in frequency when it was decreased in the LV, regardless of the other parameters changed. However, increasing IK1 in the myopathic RV was not always associated with a decrease in frequency. In the RV, decreasing Cx43 and IKr individually increased frequency, but combined, decreased frequency. These results indicate that the Cx43 and IKr act as molecular components that most likely alter frequency by two different mechanisms, and together the two channels have antagonistic effects on frequency. Furthermore, if the system was simply linear, the two plots would be complements of each other, i.e., increasing a parameter leading to an increase of frequency in one ventricle would lead to a decrease in frequency when the parameter is decreased in the other ventricle, resulting in blue and red being exchanged between the two plots. However, this is not the case since additional parameters were different in the RV and LV, which were not changed. Specifically, NCX and CASQ were slightly different. In the LV, the largest decrease was seen with changes in Cx43 and NaK, which corresponds to the largest frequency increase seen in the RV. However, the largest frequency increase in the LV, changing IK1 and IKr, did not correspond to the largest frequency decrease in the RV. The results of our computer simulations illustrate the complexity of determinants regulating cycle frequency. Since frequency was averaged over an area, factors, which affected it, included the absolute refractory period, the propensity for block, tissue dimensions, and the size and movement of the rotor core. The core region was important since the core itself did not demonstrate action potentials, serving to decrease average frequency. In the RV, the maximum frequency observed over the tissue was decreased for only one case (NaK−,IK1+) yet average frequencies either increased and decreased. An increase in both the minimum and maximum frequencies was observed when Cx43, NaK and IK1 were changed in the RV but an overall decrease in frequency was observed. Thus, global behavior could not be predicted by changes in one single characteristic.

## Discussion

In this study the comprehensive regional ion channel transcript profile in failing human hearts was compared to normal hearts and demonstrated significant differences in multiple transcripts. As a practical strategy, in a subset of genes we studied the protein expression levels with Western blot analysis of LV endocardial tissue from control and myopathic hearts. This analysis demonstrated differences in protein expression between control and myopathic hearts, which were concordant with the mRNA expression profiles for the genes studied. The regional differences in transcript expression were correlated to functional electrophysiological parameters during ventricular fibrillation and through use of statistical model we suggest that a limited number ion channels correlate with the heterogeneity in regional fibrillation dynamics, and may serve as targets for future studies for modulation of human VF in myopathic human hearts.

### Expression Signatures in Myopathic Hearts

Previous studies using microarrays for transcriptomal profiling have examined gene expression differences across cardiac regions in various models [18], [19], [20], [21]. The current study is confirmatory and expands these findings in humans by comparing gene expression within and between myopathic and non-diseased hearts. Further, our study differs by specifically focusing on ion channels, connexin, calcium cycling and related subunits. In comparison to these previous studies, our findings demonstrate several significant differences likely the result of the different experimental procedures and sources for tissue sampling, as well as methods and technology (TLDA) utilized. Finally, our study helps provide correlations between the various genes analyzed and the possible role they may play, thereby providing novel substrates for further investigation.

Reductions in the transient outward current I_to_ during phase 1 of the cardiac action potential prolongs action potential duration and may be proarrhythmic [22]. In the myopathic LV and RV we report significant down regulation in the subunit largely responsible for the fast phase of I_to_ (I_to,f_, Kv4.3), and dysregulation of subunits which contribute to the slow phase of I_to_ (I_to,s_, ↑Kv1.4, ↓Kv1.7). The reduction in I_to,f_ not only prolongs action potential duration but adversely modulates excitation-contraction coupling by affecting L-type Ca^2+^ magnitude [23]. Multiple animal studies have demonstrated a reduction in the I_k1_ current in the myopathic state, and related I_k1_ to VF [3], [5], [24], [25], [26]. In our model Kir 2.2 was reduced in both myopathic ventricles when compared to normal. While I_kACh_ plays a significant role in the response to vagal input and atrial fibrillation the role in human ventricular myopathy is unclear [27]. Our findings demonstrated a significant reduction in both LV and RV myopathic ventricles for Kir3.4 transcripts. Kir3.4 subunits form a heteromeric assembly with Kir3.1 subunits, which leads to the production of I_kACh_ in both ventricles, the dysregulation of Kir3.4 and its association with myopathy warrants further investigation.

Heart failure causes defective Ca^2+^ sequestration into the sarcoplasmic reticulum due to a reduction in the expression of SERCA2, which leads to reduced amplitude and slowed decay of the intracellular Ca transient [28]. Consistent with previous studies, the expression of SERCA2 was significantly lower in the myopathic compared to the normal ventricles. Slower kinetics of Ca^2+^ reuptake by the sarcoplasmic reticulum can result in an increased propensity for T-wave alternans and early after-depolarisations that are proarrhythmic [29]. Although there was no significant difference in RyR2 expression between the myopathic and normal hearts, the expression was much lower in the myopathic LV compared to the myopathic RV. Compartmental reduction of SERCA2 and RyR2 expression may contribute to arrhythmia heterogeneity with the LV being at greater risk of arrhythmia initiation on the basis of spontaneous calcium leak compared to RV ectopy. Expression of the SR calcium storage protein, calsequestrin was significantly increased in the myopathic compared to normal hearts. Increased calsequestrin is predicted to increase Ca^2+^ storage capacity of the sarcoplasmic reticulum; however, release of Ca^2+^ by the SR may be decreased [30]. While the physiological response of increased calsequestrin expression in myopathic human hearts has not been described, over expression of calsequestrin leads to hypertrophy and decreased contractility in mice [31]. Our findings provide further evidence into the potential influential role SERCA2 and RyR2 may play in VF maintenance. While these findings do not provide definitive conclusions it yields information, which warrant future investigation and study.

As it would be impractical to perform protein analysis of all 84 genes studied in the transcriptomal analysis, we performed selective Western blot analysis of important genes to verify that the comparison of normal to myopathic was indeed valid. Our protein analysis indicated that the transcriptome analysis was effective at assessing which gene products were up- or downregulated between normal and myopathic hearts. As such, provides additional support to the correlative relationships between gene expression and VF dynamics we set forth in our study.

### Transcriptomal profile and VF Dynamics

Animal studies have linked alteration of specific ionic currents, particularly I_k1_, with VF dynamics [3], [5], [24], [25], [26]. However, this paradigm was established in models where single channels/currents were considered. With the exception of specific primary arrhythmic disorders such as Brugada and long QT syndromes, disturbances in cardiac conduction and repolarisation in myopathic hearts are generally the result of altered expression of multiple ion channels and gap junction subunits [32], [33]. The alteration of one channel leads to compensatory mechanisms affecting other channels, and remodeling in disease states likely produces specific expression signatures involving the pantheon of ion channels. Though alterations of single channel provides for rigorous hypothesis testing, in the clinical myopathic state where there are changes to multiple ion channels the determinants of VF dynamics becomes more complex. The transcriptomal methodology used in this study allows portraits of global expression to be created from small samples while allowing detailed near real-time electrophysiological measurements in arrhythmia. This combined approach is critical to understanding the physiological and clinical importance of regional expression variations, which promote arrhythmogenesis in the whole organ. While our transcriptomal approach allows us to only make correlations between the genes analyzed in this study and their potential electrophysiological implications, it provides a framework to provide further understanding into the complex mechanism of arrhythmogenesis.

While our study primarily focused on characterizing the transcriptomal profile of myopathic and normal hearts, we attempted to make further correlations between specific genes identified in our analysis with VF generation. The correlative model we constructed suggests ion channel transcript expressional variability correlates to regional differences in electrophysiological parameters observed during VF. The role of I_k1_ in local VF dynamics is complex and has a varying role depending how far it is from the core of the rotor [34]. Despite this complexity, expression level of Kir2.1 and Kir2.3 were significant determinants of VF dynamics in the regression models. In murine monolayers heterogeneity of Kir2.1 expression has been demonstrated to contribute to the genesis and stability of spiral waves [5]. Thus local heterogeneity of Kir2.1 is a critical component of VF dynamics and may be more important than absolute levels in determining local cycle lengths. It has been elegantly shown that greater Ik1 expression allows for rapidity of rotational speed of rotors, as such our finding of a positive correlation with cycle length may appear contradictory. It is, however, important to note that the findings of Dr. Jalife relates to the highest frequency found at a particular point [34], and not the average over the region. The findings of Ik1 expression allowing for rapidity of rotational speed is applicable if we had removed myocardial samples at the rotor anchor points in all samples we analyzed, which is unlikely. It is most likely that we sampled away from the rotor. The distance from the rotor has an impact on whether this expression would have an increasing or decreasing effect on cycle length [34]. It has been also shown that the rectification and resting membrane potential have a significant effect on the influence of Ik1 on VF dynamics and may be pertinent to our apparent contradictory findings, especially in the myopathic hearts, where the resting membrane potential is altered [35]. To better understand the contribution of multiple ion channels changes that occur simultaneously, on VF frequency we modified the parameters of four molecular components: Cx43, hERG, Na^+^/K^+^ ATPase ß1 and Kir2.1. These components contribute to factors, which regulate rotor core movement, refractory period and conduction block. In our computer simulation average rotor frequencies calculated under the various simulations demonstrates that IK1 may play a role in either increasing or decreasing rotor frequency depending on its interplay with other channels. In the LV, decreasingIK1 was shown to consistently increase average VF frequency regardless of the contribution from any of the other channels, consistent with this study. However, the minimum and maximum frequencies observed within the tissue were decreased, keeping in line with reported effects of IK1 block on VF frequency [34].The response of changing IK1 in the RV was much more variable. While our regression model provided an insight into the role of ion channel changes on VF dynamics in human cardiomyopathy, our computer simulations provide evidence demonstrating the complex nature of how VF dynamics are regulated in opposite directions predicted by known linear relationships of single ion channel changes depending on the interplay of other determinants.

As expected, increasing expression of Na^+^/K^+^ ATPase ß1 resulted in faster conduction velocity and shorter VF cycle lengths. Increased hERG (I_kr_) resulted in decreased cycle length, possibly by increasing conduction velocity as a result of shortening repolarization time. In our model, the most significant predictor of VF cycle length at each of the three sites was the level of Cx43 expression. Surprisingly increased expression of Cx43 resulted in an increase in cycle length. Though this study cannot establish the mechanistic rationale, this may suggest the regions studied have provided pivot points for reentry and thus have registered higher activation in regions with decreased Cx43 expression, as a result of spatial heterogeneity [36], [37], [38]. Alternatively, altered post-translational modification of Cx43 (hypophosphorylation and lateralization of gap junctions) in heart failure may alter gap junction electrotonic influences on activation rates. An increase in Cx45 or Cx43 resulted in decreased conduction block. Increases in either connexin subunit alters the Cx45:Cx43 ratio reducing gap junctional inter-cellular communication which may result in increased conduction block. However, increases in homomeric homotypic Cx43 and Cx45 gap junctions may reduce functional conduction block by improving inter-cellular conduction. Overall our findings signifying the importance of connexin subunits in regulation of VF dynamics are in concordance with findings from the Efimov group [12]. They demonstrated the significance of Cx43 expression in regulating conduction velocity in ventricular wedge preparations from human hearts with end-stage nonischemic cardiomyopathy. The downregulation of Cx43 in addition to reduction in protein phosphorylation were identified as critical components in generation of arrhythmogenic states in those with nonischemic cardiomyopathy. Increasing SUR2 (I_kATP_) was also a determinant of VF dynamics, perhaps by action potential duration modulation due to global ischaemia that ensues during VF and activation of these channels shortening repolarization duration. Our group has published the importance of K_ATP_ subunit gene expression in cardiomyopathic human hearts [39]. We demonstrated heterogenous expression pattern of Kir6.1, Kir6.2, SUR1 and SUR2A throughout the left ventricle. Additionally, we showed that K_ATP_ channel blockade led to the promotion of spontaneous defibrillation in cardiomyopathic human hearts by attenuating the ischemia-dependent spatiotemporal heterogeneity of refractoriness during early VF [39]. Interestingly the gene expression data of our current study preceded the findings of our work with the K_ATP_ channel subunits. As such, the identification of genes in this present study and the strong correlative relationship characterized through our statistical model provides promise for the use of our shotgun approach in the further identification of ion channel variation which may play a key contributory role in VF dynamics.

### Limitations

In this study, we examined alterations in mRNA expression levels of various ion channel transcripts in the septum, RV and LV. There is no mRNA-based assay that provides information on mRNA stability, posttranslational processes, or subunit assembly. Therefore, mRNA transcript levels may not correspond directly to channel activity. The goal of this study was to utilize a shot-gun gene analysis approach to identify as many potentially novel molecular targets involved in VF dynamics, such that future ion channel specific studies can be formulated with the channels identified. While we acknowledge that definitive correlations on VF dynamics cannot be made unless channel activity can be isolated for all 84 ion channels studies, it nevertheless provides a compilation of potential targets, which will be the focus of future studies. These studies will be focused on individual molecular targets identified within this study and will include cell isolation and patch clamp experiments. Local VF cycle length and conduction block is also determined by local myocardial architecture, particularly the amount of scar/fibrosis. Our previous study of VF dynamics evaluating the role of anatomic structure suggested that regional VF dynamics are only partially explained by such differences [10]. As such, the focus of this study was to determine the spatial gene expression profile of ion channel subunits in normal compared to myopathic tissue samples, and to then use this data in combination with regression analysis to generate correlations which could be used to yield insight into the molecular determinants which may contribute to VF dynamics.

Samples were taken from areas of visually and palpably healthy myocardium in order to isolate ion channel remodeling effects, from local fibrosis, on VF dynamics. Additionally, we were not able to fully eliminate the possibility of contamination by purkinje fibers in our isolated endocardial biopsies. Finally, we acknowledge the heterogeneity of the myopathic hearts used in this study. The different disease states among the myopathic hearts may lead to undetermined differences in ion channel expression and VF dynamics which were not examined separately in this study.

To study fibrillation dynamics, we used VFCL as a surrogate for refractoriness during VF as previously published (28). In this study we did not specifically evaluate regional electrical remodeling as measured by APD and CV and its relationship to VF dynamics. This was the basis of detailed studies conducted by Nanthakumar et al [2], [40]. In those studies of an in-vivo model of VF, using 1008 electrodes with 2 mm resolution, regional electrical properties were related to regional DF, activation maps, excitable gap and reentry. However in that study, the influence of regional molecular characteristics to regional VF dynamics was not performed and was the basis of the design of this study.

## Conclusions

Ion channel expression profile in myopathic human hearts is significantly altered compared to normal hearts and reveals regional differences. The correlative relationships between several specific genes with VF dynamics and the modeling studies indicate a complex interactive influence on cycle length and conduction block. Our study provides an expression profile of molecular targets, which may contribute to VF within myopathic hearts. Using regression analysis and computer simulations we have uncovered complex interactions of key ion channel determinants of VF dynamics that could provide novel substrates for safe therapeutic strategies.

## Supporting Information

Table S1 List of genes analyzed using the TaqMan low-density gene arrays. The Gene ID numbers are provided along with the identification for each reference probe used in the analysis.
(PDF)

Table S2 Expression profile data for all genes analyzed. Gene name and corresponding protein expressed are listed. The genes are grouped according to different families of ion channels. Raw expression values for normal and myopathic LV and RV are listed, along with N and SEM.
(PDF)
