# Supplementary material for: Regional Ion Channel Gene Expression Heterogeneity and Ventricular Fibrillation Dynamics in Human Hearts
Source: PLoS One. 2014 Jan 10;9(1):e82179. doi: 10.1371/journal.pone.0082179 (PMC3888386; doi:10.1371/journal.pone.0082179)
Supplement: Table S1 — List of genes analyzed using the TaqMan low-density gene arrays. The Gene ID numbers are provided along with the identification for each reference probe used in the analysis. (PDF) [file pone.0082179.s001.pdf]

Table 1. List of genes analyzed using the TaqMan Low Density Arrays.

GENE ID	Reference Probe Applied Biosystem
ABCC8	Hs00165861_m1
ABCC9	Hs00245832_m1
ATP1A1	Hs00167556_m1
ATP1A3	Hs00265163_m1
ATP1B1	Hs00426868_g1
ATP2A2	Hs00155939_m1
ATP2A3	Hs00193090_m1
ATP2B1	Hs00155949_m1
ATP2B4	Hs00608066_m1
CACNA1C	Hs00167681_m1
CACNA1D	Hs00167753_m1
CACNA1G	Hs00367969_m1
CACNA1H	Hs00234934_m1
CACNA2D1	Hs00167808_m1
CACNA2D2	Hs00195772_m1
CACNB2	Hs00167861_m1
CACNG4	Hs00205244_m1
CACNG6	Hs00230428_m1
CALM1	Hs00300085_s1
CALM3	Hs00270914_m1
CASQ1	Hs00154281_m1
CASQ2	Hs00415779_m1
CD4	Hs00181217_m1
CFTR	Hs00357011_m1
CLCN2	Hs00189078_m1
CLCN3	Hs00156527_m1
CLCN6	Hs00154518_m1
CLCN7	Hs00241850_m1
CNN1	Hs00154543_m1
COL6A1	Hs00242448_m1
G6PD	Hs00166169_m1
GJA1	Hs00748445_s1
GJA5	Hs00270952_s1
GJA7	Hs00271416_s1
HCN1	Hs00395037_m1
HCN2	Hs00606903_m1
HCN3	Hs00380018_m1
HCN4	Hs00175760_m1
HPRT1	Hs99999909_m1
IL6	Hs00174131_m1
ISYNA1	Hs00375021_g1
ITPR1	Hs00181881_m1
ITPR3	Hs00609908_m1
KCNA1	Hs00264798_s1
KCNA2	Hs00270656_s1
KCNA3	Hs00704943_s1
KCNA4	Hs00357903_s1
KCNA5	Hs00266898_s1
KCNA6	Hs00266903_s1
KCNA7	Hs00361015_m1
KCNAB1	Hs00185764_m1
KCNAB2	Hs00186308_m1
KCNAB3	Hs00190986_m1

KCNB1	Hs00270657_m1
KCNC3	Hs00192108_m1
KCNC4	Hs00428198_m1
KCND1	Hs00192113_m1
KCND2	Hs00273378_m1
KCND3	Hs00542593_m1
KCNE1	Hs00264799_s1
KCNE1L	Hs00273381_s1
KCNE2	Hs00270822_s1
KCNE3	Hs00538801_m1
KCNE4	Hs00758199_g1
KCNH2	Hs00165120_m1
KCNIP2	Hs00601709_g1
KCNJ11	Hs00265026_s1
KCNJ12	Hs00266926_s1
KCNJ2	Hs00265315_m1
KCNJ3	Hs00158421_m1
KCNJ4	Hs00705379_s1
KCNJ5	Hs00168476_m1
KCNJ8	Hs00270663_m1
KCNK1	Hs00158428_m1
KCNK3	Hs00605529_m1
KCNK5	Hs00186652_m1
KCNQ1	Hs00165003_m1
MYH7	Hs00165276_m1
NPPA	Hs00383230_g1
NPPB	Hs00173590_m1
PIAS3	Hs00180666_m1
PLN	Hs00160179_m1
PPP3CA	Hs00174223_m1
RYR2	Hs00181461_m1
SCN1A	Hs00374696_m1
SCN1B	Hs00168897_m1
SCN2B	Hs00394952_m1
SCN3A	Hs00366902_m1
SCN3B	Hs00393218_m1
SCN4A	Hs00165686_m1
SCN5A	Hs00165693_m1
SCN7A	Hs00161546_m1
SCN9A	Hs00161567_m1
SLC8A1	Hs00253432_m1
UCHL1	Hs00188233_m1
