# Supplementary material for: Regional Ion Channel Gene Expression Heterogeneity and Ventricular Fibrillation Dynamics in Human Hearts
Source: PLoS One. 2014 Jan 10;9(1):e82179. doi: 10.1371/journal.pone.0082179 (PMC3888386; doi:10.1371/journal.pone.0082179)
Supplement: Table S2 — Expression profile data for all genes analyzed. Gene name and corresponding protein expressed are listed. The genes are grouped according to different families of ion channels. Raw expression values for normal and myopathic LV and RV are listed, along with N and SEM. (PDF) [file pone.0082179.s002.pdf]

Expression profile of for all genes analyzed

Genes	Proteins	Mean LV Normal	N	SEM	Mean RV Normal	N	SEM	Mean LV Myopathic	N	SEM	Mean RV Myopathic	N	SEM	Mean Septum Myopathic	N	SEM
Sodium channels and connexins																
SCN1A	Nav1.1	1,78	8	*0,77*	1,22	8	*0,36*	0,74	7	*0,15*	1,02	8	*0,20*	0,51	7	*0,14*
SCN3A	Nav1.3	3,51	8	*0,78*	3,84	8	*0,89*	6,80	8	*1,02*	6,73	8	*0,50*	5,59	8	*0,58*
SCN5A	Nav1.5	443,38	8	*65,23*	381,81	8	*36,62*	457,15	8	*20,26*	426,37	8	*23,17*	471,93	8	*33,77*
SCN7A	Nav2.1	220,88	8	*22,41*	299,60	8	*26,94*	380,50	8	*53,26*	338,65	8	*15,14*	351,34	8	*55,55*
SCN9A	Nav1.7	4,89	8	*0,47*	6,56	8	*0,67*	3,59	8	*0,57*	4,04	8	*0,42*	2,89	8	*0,42*
SCN1B	Navβ1	77,40	8	*10,77*	90,07	8	*10,25*	102,53	8	*10,12*	91,05	8	*5,44*	94,46	8	*9,85*
SCN2B	Navβ2	33,40	8	*8,42*	27,00	8	*6,45*	74,81	8	*3,48*	61,77	8	*4,40*	81,03	8	*8,28*
SCN3B	Navβ3	3,80	8	*0,67*	3,78	8	*0,47*	4,43	8	*0,57*	3,59	8	*0,42*	5,10	8	*0,48*
GJA1	Cx43	1603,81	8	*183,32*	1462,36	8	*76,73*	1317,86	8	*127,80*	1661,47	8	*125,65*	1294,59	8	*94,49*
GJA5	Cx40	31,53	8	*3,58*	32,75	8	*3,54*	49,85	8	*14,52*	40,96	8	*9,20*	25,21	8	*2,78*
GJA7	Cx45	136,88	8	*26,32*	109,88	8	*11,61*	102,78	8	*4,96*	91,32	8	*6,08*	92,07	8	*7,92*
Calcium channels																
CACNA1C	Cav1.2	518,17	8	*61,62*	491,39	8	*46,50*	560,97	8	*39,97*	543,46	8	*29,72*	573,95	8	*32,12*
CACNA1D	Cav1.3	0,40	8	*0,09*	0,31	8	*0,06*	0,43	8	*0,09*	0,41	8	*0,07*	0,48	7	*0,08*
CACNA1G	Cav3.1	0,37	6	*0,08*	0,56	7	*0,16*	0,57	8	*0,09*	1,21	8	*0,27*	0,69	7	*0,15*
CACNA1H	Cav3.2	3,49	8	*0,26*	6,07	8	*0,58*	8,08	8	*1,51*	6,77	8	*1,21*	6,80	8	*1,75*
CACNA2D1	Cavα2δ1	159,86	8	*15,38*	131,48	8	*8,85*	236,21	8	*15,84*	216,61	8	*14,74*	243,55	8	*19,47*
CACNA2D2	Cavα2δ2	22,29	8	*2,94*	22,66	8	*1,53*	21,45	8	*2,00*	26,76	8	*2,14*	35,22	8	*2,62*
CACNB2	Cavβ2	137,01	8	*24,42*	126,92	8	*19,92*	94,08	8	*12,68*	149,22	8	*6,09*	117,24	8	*13,90*
Cl^- and HCN channels																
CLCN2	ClC-2	3,86	8	*0,27*	5,81	8	*0,75*	4,85	8	*0,44*	4,82	8	*0,64*	4,38	8	*0,47*
CLCN3	ClC-3	165,24	8	*11,86*	198,62	8	*14,47*	181,30	8	*12,90*	195,59	8	*10,27*	194,48	8	*12,58*
CLCN6	ClC-6	114,25	8	*5,74*	127,80	8	*5,96*	114,33	8	*3,00*	114,80	8	*5,02*	118,15	8	*4,50*
CLCN7	ClC-7	58,06	8	*4,26*	93,32	8	*13,89*	55,80	8	*3,71*	53,16	8	*3,85*	57,32	8	*5,04*
CFTR	Cftr	2,27	6	*0,64*	2,48	8	*0,53*	0,15	5	*0,04*	0,32	6	*0,14*	0,26	4	*0,11*
HCN1	Hcn1	0,78	7	*0,23*	0,61	8	*0,18*	0,45	7	*0,10*	0,68	7	*0,18*	0,63	8	*0,12*
HCN2	Hcn2	46,00	8	*8,70*	9,24	8	*1,32*	69,27	8	*14,98*	19,78	8	*5,76*	46,84	8	*14,88*
HCN3	Hcn3	3,57	8	*0,35*	4,43	8	*0,67*	3,09	8	*0,39*	2,75	8	*0,21*	2,61	8	*0,26*
HCN4	Hcn4	64,18	8	*6,03*	73,05	8	*9,05*	42,93	8	*8,25*	43,08	8	*7,48*	44,87	8	*9,91*
Pumps, Exchangers and Calcium handling proteins																
ATP1A1	Na/K-ATPase, α1	369,18	8	*66,07*	330,81	8	*26,32*	241,06	8	*13,72*	265,92	8	*23,97*	253,05	8	*22,00*
ATP1A3	Na/K-ATPase, α3	1599,52	8	*146,13*	1769,95	8	*97,85*	1128,90	8	*70,86*	1277,98	8	*91,14*	1038,07	8	*43,65*
ATP1B1	Na/K-ATPase, β1	1119,32	8	*74,20*	997,70	8	*70,33*	836,58	8	*30,03*	933,03	8	*50,29*	846,78	8	*49,24*
SLC8A1	NCX1	755,96	8	*62,34*	625,55	8	*49,09*	725,31	8	*34,19*	762,10	8	*25,93*	804,68	8	*32,48*
ATP2A2	SERCA2	4635,35	8	*333,73*	4827,01	8	*306,21*	2214,78	8	*160,50*	2863,62	8	*253,89*	2268,84	8	*274,19*
ATP2A3	SERCA3	14,96	8	*1,44*	15,92	8	*1,48*	13,06	8	*1,34*	11,32	8	*1,42*	10,67	8	*1,38*
ATP2B1	PMCA1	40,82	8	*8,88*	37,99	8	*2,58*	42,82	8	*3,07*	38,37	8	*3,14*	43,69	8	*5,92*
ATP2B4	PMCA4	385,54	8	*43,57*	321,96	8	*25,66*	329,00	8	*25,33*	303,59	8	*30,62*	370,20	8	*32,44*
PLN	PLB	16292,37	8	*1380,77*	14969,11	8	*2155,61*	8658,84	8	*460,77*	9954,70	8	*593,50*	9332,40	8	*755,24*
CALM1	Calm1	680,32	8	*56,70*	532,71	8	*44,55*	541,08	8	*58,43*	555,46	8	*51,47*	503,43	8	*46,64*
CALM2	Calm2	143,20	4	*23,04*	166,26	5	*23,79*	114,89	8	*7,74*	120,36	8	*11,14*	95,32	8	*6,94*
CALM3	Calm3	1912,21	8	*101,56*	2080,85	8	*95,53*	1790,97	8	*164,24*	1961,22	8	*66,21*	2042,28	8	*103,35*
CASQ1	Casq1	117,13	8	*29,65*	57,67	8	*6,40*	92,72	8	*19,49*	78,15	8	*18,08*	135,90	8	*17,91*
CASQ2	Casq2	2108,07	8	*90,71*	2032,56	8	*84,64*	2671,54	8	*121,65*	2809,94	8	*170,95*	3108,64	8	*120,39*
ITPR1	RIP3-1	62,17	8	*5,13*	76,11	8	*5,92*	66,93	8	*4,76*	55,36	8	*4,40*	57,88	8	*4,53*
ITPR3	RIP3-3	21,63	8	*3,39*	29,25	8	*3,88*	22,43	8	*1,50*	20,90	8	*1,93*	21,34	8	*2,38*
RYR2	RYR2	5711,99	8	*222,11*	5557,79	8	*317,37*	5502,88	8	*325,92*	6686,84	8	*471,68*	5866,32	8	*360,51*
PPP3CA	CAM-PRP α	63,82	8	*4,73*	81,25	8	*11,71*	53,66	8	*3,33*	54,82	8	*3,90*	52,48	8	*2,77*
PPP3CB	CAM-PRP β	310,88	4	*11,20*	336,27	5	*27,09*	373,01	8	*25,42*	354,87	8	*17,45*	368,98	8	*16,39*
PPP3CC	CAM-PRP γ	117,08	4	*18,30*	123,96	5	*13,17*	100,90	8	*9,96*	98,67	8	*7,99*	99,75	8	*6,37*

Genes	Proteins	Mean LV Normal	N	SEM	Mean RV Normal	N	SEM	Mean LV Myopathic	N	SEM	Mean RV Myopathic	N	SEM	Mean Septum Myopathic	N	SEM
Kv and Kir channels	***α-subunits and K+ β-subunits***															
KCNA2	Kv1.2	2,17	8	*0,36*	4,18	8	*0,68*	2,91	8	*0,48*	4,70	8	*1,05*	2,90	8	*0,64*
KCNA3	Kv1.3	1,91	4	*0,88*	1,32	4	*0,49*	1,44	8	*0,22*	1,58	8	*0,24*	1,47	8	*0,36*
KCNA4	Kv1.4	11,64	8	*1,82*	8,43	8	*0,87*	21,19	8	*1,20*	20,35	8	*2,32*	26,03	8	*2,42*
KCNA5	Kv1.5	12,55	8	*1,18*	18,59	8	*2,38*	17,99	8	*1,63*	15,88	8	*1,46*	14,47	8	*1,88*
KCNA6	Kv1.6	3,06	8	*1,01*	3,56	8	*0,74*	4,01	8	*0,33*	3,40	8	*0,30*	2,90	8	*0,68*
KCNA7	Kv1.7	5,40	8	*1,27*	5,77	8	*1,25*	0,81	8	*0,22*	0,69	8	*0,33*	0,64	8	*0,20*
KCNB1	Kv2.1	7,69	8	*2,19*	8,95	8	*2,98*	12,62	8	*1,94*	14,28	8	*2,26*	10,34	8	*1,31*
KCNC3	Kv3.3	2,70	8	*0,36*	2,18	8	*0,24*	1,87	8	*0,31*	1,52	8	*0,16*	1,58	8	*0,11*
KCNC4	Kv3.4	10,66	8	*1,19*	14,80	8	*1,98*	17,23	8	*1,36*	15,35	8	*1,92*	15,71	8	*1,49*
KCND1	Kv4.1	0,82	8	*0,13*	1,50	8	*0,26*	1,18	8	*0,19*	1,13	8	*0,13*	0,89	8	*0,16*
KCND2	Kv4.2	0,70	8	*0,11*	0,83	8	*0,23*	1,44	8	*0,65*	1,30	8	*0,51*	1,46	8	*0,54*
KCND3	Kv4.3	26,72	8	*1,60*	26,07	8	*1,73*	16,30	8	*0,91*	18,17	8	*1,62*	15,46	8	*1,25*
KCNH2	Herg	191,23	8	*17,96*	195,47	8	*14,86*	105,68	8	*5,08*	115,55	8	*3,47*	107,41	8	*6,44*
KCNQ1	KvLQT1	80,11	8	*6,54*	91,47	8	*3,27*	66,65	8	*4,58*	63,01	8	*3,23*	71,01	8	*4,31*
KCNK1	TWIK1	68,62	8	*13,44*	80,93	8	*8,82*	52,46	8	*10,32*	52,64	8	*7,18*	51,97	8	*4,99*
KCNK3	TASK	2,37	8	*0,63*	2,65	8	*0,58*	2,42	8	*0,67*	1,37	8	*0,38*	1,14	8	*0,30*
KCNK5	TASK2	1,50	8	*0,35*	2,27	8	*0,47*	1,07	8	*0,27*	0,97	8	*0,18*	1,00	8	*0,23*
KCNJ2	Kir2.1	134,46	8	*17,89*	95,99	8	*12,98*	162,89	8	*18,91*	169,16	8	*29,44*	175,72	8	*22,63*
KCNJ3	Kir3.1	3,26	8	*0,75*	7,24	8	*3,73*	5,23	8	*1,38*	3,70	7	*1,41*	2,75	8	*1,04*
KCNJ4	Kir2.3	80,89	8	*10,26*	46,48	8	*6,30*	132,44	8	*12,81*	84,15	8	*8,27*	139,04	8	*13,70*
KCNJ5	Kir3.4	15,32	8	*1,34*	20,90	8	*2,95*	10,17	8	*2,01*	12,32	8	*2,22*	17,40	8	*2,21*
KCNJ8	Kir6.1	186,21	8	*18,51*	272,73	8	*36,51*	184,55	8	*20,46*	192,65	8	*15,54*	189,02	8	*17,30*
KCNJ11	Kir6.2	36,38	8	*5,13*	41,44	8	*6,10*	26,37	8	*2,22*	27,26	8	*2,26*	24,33	8	*1,69*
KCNJ12	Kir2.2	74,62	8	*8,69*	90,30	8	*9,83*	46,42	8	*2,16*	53,12	8	*2,47*	54,21	8	*3,54*
KCNAB1	Kvβ1	9,53	8	*1,53*	10,66	8	*1,04*	19,19	8	*1,87*	16,91	8	*1,50*	16,44	8	*2,02*
KCNAB2	Kvβ2	139,97	8	*18,59*	115,73	8	*16,79*	155,05	8	*33,92*	120,32	8	*19,71*	136,87	8	*27,06*
KCNAB3	Kvβ3	0,76	8	*0,16*	0,77	8	*0,21*	0,46	8	*0,10*	0,51	8	*0,07*	0,48	8	*0,07*
KCNIP2	KChIP2	222,26	8	*33,29*	320,57	8	*22,74*	47,73	8	*11,61*	119,68	8	*25,22*	62,27	8	*26,97*
KCNE1	MinK	14,74	8	*1,94*	11,80	8	*0,99*	17,80	8	*2,43*	16,82	8	*1,83*	18,53	8	*2,16*
KCNE1L	MIRP4	0,58	8	*0,13*	1,03	8	*0,16*	0,85	8	*0,11*	0,82	8	*0,16*	1,12	8	*0,22*
KCNE2	MIRP1	1,14	8	*0,18*	1,08	8	*0,24*	1,15	8	*0,16*	1,42	8	*0,24*	1,08	8	*0,16*
KCNE3	MIRP2	3,95	8	*0,53*	5,35	8	*0,70*	3,70	8	*0,42*	4,05	8	*0,50*	3,52	8	*0,29*
KCNE4	MIRP3	6,74	8	*0,88*	8,90	8	*1,12*	14,39	8	*5,65*	19,85	8	*8,51*	10,60	8	*4,06*
ABCC8	SUR1	4,11	8	*0,49*	3,84	8	*0,84*	7,92	8	*1,66*	8,26	8	*1,57*	7,21	8	*2,05*
ABCC9	SUR2	227,35	8	*19,54*	259,08	8	*16,30*	251,09	8	*18,28*	292,98	8	*23,03*	220,34	8	*8,60*
PIAS3	KChAP	17,07	8	*1,22*	17,38	8	*1,19*	17,93	8	*0,80*	16,21	8	*0,70*	16,31	8	*1,02*
